# The mediating role of psychological contract breach in the relationship between illegitimate tasks and turnover intention among Chinese nurses: a cross-sectional study

**DOI:** 10.3389/fpsyg.2025.1712653

**Published:** 2026-01-12

**Authors:** Tianyu Yin, Wenting He, Yao Xie, Jing Ma, Xiuting Liao

**Affiliations:** Department of Obstetrics and Gynecology, Sichuan Provincial People’s Hospital, School of Medicine, University of Electronic Science and Technology of China, Chengdu, China

**Keywords:** cross-sectional study, illegitimate tasks, nurses, psychological contract breach, turnover intention

## Abstract

**Background:**

Nurse shortage and workforce turnover have become critical issues in the global public health sector, recognized internationally as priority concerns. This challenge is particularly prominent in China, where the dual pressures of nurse shortage and high turnover are more acute. Optimizing nursing management processes and creating a highly supportive work environment can effectively alleviate the psychological resource depletion and reduced job satisfaction caused by stress among nurses, thereby lowering their turnover intention. However, research on how illegitimate tasks influence the turnover intention of Chinese nurses remains scarce, and the mediating mechanism of psychological contract breach in this relationship lacks sufficient context-specific verification.

**Objective:**

This study aims to explore the direct relationships among illegitimate tasks, psychological contract breach, and turnover intention among Chinese clinical nurses, and specifically examine the mediating role of psychological contract breach in the relationship between illegitimate tasks and turnover intention among nurses.

**Methods:**

A cross-sectional study design was employed. From March 2024 to May 2025, a convenience sampling method was used to select 516 clinical nurses from Sichuan Province for a survey. The survey included a demographic information questionnaire, the Illegitimate Tasks Scale, the Psychological Contract Breach Scale, and the Turnover Intention Scale.

**Results:**

The results showed that the score of turnover intention among clinical nurses was (13.14 ± 4.15), with 71 nurses (13.7%) having a very high turnover intention. The score of psychological contract breach was (55.79 ± 15.50), and the score of illegitimate tasks was (29.06 ± 8.69). Correlation analysis revealed that the total score of illegitimate tasks and its dimensions were positively correlated with the total score of psychological contract breach and its dimensions (*r* = 0.401–0.623, *p* < 0.01), as well as with the total score of turnover intention and its dimensions (*r* = 0.446–0.495, *p* < 0.01). The total score of psychological contract breach and its dimensions were also positively correlated with the total score of turnover intention and its dimensions (*r* = 0.439–0.733, *p* < 0.01). Mediating effect analysis indicated that psychological contract breach played a significant mediating role between illegitimate tasks and turnover intention, with a mediating effect value of 0.153, accounting for 64.8% of the total effect.

**Conclusion:**

This study confirms that psychological contract breach mediates the relationship between illegitimate tasks and turnover intention among nurses. Reducing psychological contract breach through interventions that decrease illegitimate tasks can effectively lower nurses’ turnover intention. Specific measures include creating a supportive work environment, establishing a sound communication mechanism, and fostering a sense of professional mission.

## 1 Background

As one of the main providers of healthcare services, nurses are an indispensable part of the human capital in hospitals and play a crucial role in promoting human health. However, against the backdrop of the growing global health demand, nurses face severe challenges such as cumbersome work tasks, insufficient career development, meager income, and inadequate mental health support. These pressures are seriously damaging the physical and mental health and job satisfaction of nurses. When nurses experience job burnout due to the aforementioned reasons, it often leads to psychological contract breach, shaking their organizational beliefs and generating turnover intention (Fang et al., 2023). A survey involving 61,168 nurses from 12 countries in the United States and Europe showed that the proportion of nurses planning to leave their jobs within 1 year ranged from 14 to 49% (Aiken et al., 2012). This phenomenon is particularly prominent in China, where 20.2–56.1% of clinical nurses have turnover intention (Li et al., 2020). Turnover intention is an important antecedent variable of turnover behavior. A 12 month study by Allen et al. (2015) on employee turnover intention and actual turnover found a positive correlation between turnover intention and actual turnover. In addition, studies have shown that a high turnover intention among nurses leads to instability in the nursing team and a shortage of nursing resources, which seriously affects the quality of nursing care (Warden et al., 2021). Therefore, exploring the influencing factors and mechanisms of nurses’ turnover intention is of great practical significance for reducing the nurse turnover rate and ensuring the quality of medical care—both scholarly (advancing nursing management theory) and professionally (guiding practical interventions).

Illegitimate tasks refer to work tasks that are inconsistent with employees’ job responsibilities, contrary to their expected work content, or not worth the time employees spend on completing them (Semmer et al., 2015). Specifically, illegitimate tasks include two categories: unreasonable tasks and unnecessary tasks. Unreasonable tasks are those that are not suitable to be assigned to specific individuals (e.g., asking nurses to repair desks and chairs, or assigning tasks requiring extensive experience to newly recruited staff). Unnecessary tasks are those that are meaningless and should not be performed at all (e.g., filing newspapers that no one will read). This situation not only violates the contract between the two parties but also breaks the psychological needs of employees, thereby causing negative emotions and attitudes. The Conservation of Resources Theory points out that the depletion of individuals’ internal resources is closely related to work motivation and well-being (Obeng et al., 2025). When nurses continuously consume their limited emotional and cognitive resources to cope with the pressure brought by illegitimate tasks, their internal psychological coping resources are exhausted, leading to a lack of identification with their work. A large number of studies have shown that illegitimate tasks can bring about various negative effects, such as evoking negative emotions in employees (Zhou et al., 2018), harming individuals’ self-esteem (Schulte-Braucks et al., 2018), reducing professional identity (Ma and Peng, 2019), inducing workplace deviant behaviors (Semmer et al., 2010), and lowering retention intention (Van Schie et al., 2014). This means that illegitimate tasks have become an important factor affecting employees’ work status and the stability of their career development. In addition, a Chinese study also showed that there is a significant correlation between illegitimate tasks and turnover intention (*r* = 0.507, *p* < 0.01) (Yu et al., 2022). However, few studies have explored the mediating mechanisms underlying this relationship, especially in the Chinese context. This indicates that the concept of illegitimate tasks has become an important research topic in the nursing field and deserves the attention of nursing managers, who should take active intervention measures to improve the situation.

The Social Exchange Theory states that the communication and interaction between individuals and organizations are effectively carried out with the goal of obtaining rewards and tangible benefits, and have a certain utilitarian nature. Fair and effective exchange behaviors maintain the gradual emergence of trust, loyalty, and commitment among the participating subjects (Homans, 1958). As a phenomenon where work content is inconsistent with job responsibilities, illegitimate tasks can be regarded as a behavior that significantly consumes psychological resources and affects the establishment and maintenance of psychological contracts. Rousseau (1989) first proposed the concept of psychological contract in 1989, which refers to an individual’s understanding and belief of the mutual obligations between the employer and the employee in the context of the employment relationship. When an individual subjectively believes that the promised obligations of the other party in the psychological contract have not been fulfilled, psychological contract breach occurs. Nursing work not only requires maintaining the “doctor-nurse-patient” relationship but also undertaking work such as reasonable charging, consumable management, equipment management, and ward management. This makes nurses undertake a large number of illegitimate tasks beyond their professional scope, leading to burnout and depletion of psychological resources. Studies have shown that psychological contract breach can lead to individuals’ self-depletion, especially when the information received (the actual performance of the organization) is inconsistent with the information conveyed (the subjective psychological contract). This will further increase the cognitive burden of employees, consume their psychological resources, generate negative emotional reactions, and further affect their work attitudes and work behaviors (Robinson and Morrison, 2000). Some scholars also believe that the subjective perception nature of psychological contracts makes psychological contract breach an unavoidable state (Restubog et al., 2015). When nurses are forced to perform illegitimate tasks for a long time, such as engaging in work unrelated to nursing, their psychological contracts will conflict due to the lack of organizational responsibility, increased work pressure, and delayed completion of scheduled work tasks, leading to psychological contract breach. Conversely, long-term psychological contract breach stimulates negative emotions such as dissatisfaction, disappointment, and regret among nurses, thereby promoting the emergence of low professional identity and turnover intention. Therefore, effectively reducing illegitimate tasks can not only effectively reduce the consumption of psychological resources but also enhance the adhesion between nurses and the organization and lower turnover intention.

The Leader-Member Exchange Theory, based on the principle of reciprocity, holds that when the employer’s behavior is in line with the interests of both labor and management, and the employee receives corresponding benefits, the employee will show behaviors and attitudes that are positive for the employer and the organization in return (Gerstner and Day, 1997). From this perspective, the superior-subordinate relationship is essentially a contractual relationship (Coyle-Shapiro et al., 2019). When the contractual relationship is broken due to the long-term imbalance of work tasks, it will cause individuals to be dissatisfied and disappointed with their work, generate negative behaviors such as work anger, avoidance, and withdrawal, and thus have turnover intention. Therefore, we have two research questions: First, what are the direct relationships among illegitimate tasks, psychological contract breach, and turnover intention among Chinese clinical nurses? Second, in this population, does psychological contract breach mediate the relationship between illegitimate tasks and turnover intention? Based on this, this study takes the Leader-Member Exchange Theory as the basis to explore the impact of illegitimate tasks on nurses’ turnover intention and the mediating role of psychological contract breach, aiming to provide a theoretical basis for improving nurses’ physical and mental health and promoting the stability of the nursing team.

Based on the above findings, this study proposes the following four hypotheses:

*Hypothesis 1*: Illegitimate tasks are positively correlated with turnover intention.

*Hypothesis 2*: Psychological contract breach is positively correlated with turnover intention.

*Hypothesis 3*: Illegitimate tasks are positively correlated with psychological contract breach.

*Hypothesis 4*: Among the nurse group, psychological contract breach plays a mediating role between illegitimate tasks and turnover intention.

## 2 Methods

### 2.1 Research purpose and design

This study employed a cross-sectional, explanatory survey design to examine the causal relationships between illegitimate tasks, psychological contract breach, and turnover intention. The purpose was to test a theoretical model that explains how illegitimate tasks influence turnover intention through psychological contract breach.

### 2.2 Participants

From March 2024 to May 2025, a convenience sampling method was used to select 516 clinical nurses from Sichuan Province for a questionnaire survey. Inclusion criteria: (1) Have obtained a nurse qualification certificate and completed practice registration in the surveyed hospital; (2) Have been engaged in clinical nursing work for ≥3 months; (3) Have been informed of the study and voluntarily participated. Sichuan Province was selected because it is a populous province with diverse healthcare settings (tertiary, secondary, and primary hospitals), and its nursing workforce faces typical challenges (e.g., staff shortages, heavy workloads, “valuing medicine over nursing” attitudes) that are representative of Chinese nurses. Exclusion criteria: Nurses on leave during the survey period.

The sample size was calculated using the formula *n* = number of independent variables × (5–10). There were 15 independent variables in this study. Considering a 20% invalid questionnaire rate, the minimum sample size was 15 × (5–10) × (1 + 20%) = 90–180 (Ni et al., 2010). A total of 516 samples were finally analyzed in this study. The study was approved by the Ethics Committee of the corresponding institution. This study has been approved by the Medical Ethics Committee of Sichuan Provincial People’s Hospital, with the approval number: Lun Shen (Yan) 2023 No. 266.

### 2.3 Study methods

#### 2.3.1 Research instruments

##### 2.3.1.1 General information questionnaire

Developed by the research team after reviewing literature and conducting discussions, it included items such as nurses’ age, gender, marital status, professional title, employment type, educational background, personal monthly income, number of night shifts per week, weekly working hours, and whether there were tasks beyond clinical work.

##### 2.3.1.2 Psychological contract breach scale

Compiled by Chinese scholar Chen (2008), this scale has been widely used in surveys of nurses. Hu et al. (2023) found that the Cronbach’s *α* coefficient of this scale was 0.963 in a survey of nurses. From the perspective of nurses, this survey explored the impact of psychological contract breach on nursing staff. The scale consists of 3 dimensions, including developmental responsibility (6 items), team responsibility (8 items), and realistic responsibility (6 items), with a total of 20 items. A 5-point Likert scale was used, with scores ranging from 1 (“strongly consistent”) to 5 (“strongly inconsistent”). The total score ranges from 20 to 100, and a higher score indicates a more severe psychological contract breach among nurses. The Cronbach’s *α* coefficient of this scale in this study was 0.944.

##### 2.3.1.3 Illegitimate tasks scale

Compiled by Chinese scholars Ge et al. (2022), this scale has been used to measure the current situation of illegitimate tasks undertaken by nurses. Zhang et al. (2025) found that the Cronbach’s *α* coefficient of this scale was 0.963 in a survey of nurses. The scale includes 2 dimensions, namely unreasonable tasks (4 items) and unnecessary tasks (4 items), with a total of 8 items. A 5-point Likert scale was used, with scores ranging from 1 (“strongly consistent”) to 5 (“strongly inconsistent”). The total score ranges from 8 to 40, and a higher score indicates that nurses undertake more illegitimate tasks. The Cronbach’s *α* coefficient of this scale in this study was 0.965.

##### 2.3.1.4 Turnover intention scale

Compiled by Michaels and Spector (1982) and translated by Chinese scholars Li and Li (2003), this scale has been widely used to measure the level of nurses’ turnover intention. Chinese scholars found that the Cronbach’s *α* coefficient of this scale was 0.975 in a survey of nurses (Liu et al., 2025). The scale includes 3 dimensions, namely turnover intention I: possibility of resignation (2 items), turnover intention II: motivation to find another job (2 items), and turnover intention III: possibility of obtaining another job (2 items), with a total of 6 items. A 4-point Likert scale was used, with scores ranging from 1 (“never”) to 4 (“often”). The total score ranges from 6 to 24, and a higher score indicates a stronger turnover intention of nurses. The evaluation criteria are as follows: the turnover intention is divided into 4 levels according to the total average score: ≤1 point (very low turnover intention), ≤2 points (low), ≤3 points (high), and >3 points (very high). The Cronbach’s *α* coefficient of this scale in this study was 0.970.

#### 2.3.2 Survey methods

Time horizon: This is a cross-sectional study with a single data collection point, reflecting nurses’ perceptions and attitudes toward their work status at the time of the survey. This study conducted an online survey via wjx.cn (an online survey platform in China) through three steps: (1) The research team compiled the questionnaire based on the purpose and content of the survey, including the purpose, content, significance of the survey, notes for filling in, and informed consent, and then uploaded the questionnaire to wjx.cn; (2) The research team contacted the relevant person in charge of the selected hospitals, identified them as investigators, and provided them with homogeneous training. The investigators sent the questionnaire link and QR code to the WeChat groups of the departments and informed the nurses of the matters related to filling in the questionnaire; (3) Eligible participants filled in the questionnaire voluntarily. All items must be completed before submission, and each IP address could only be used to fill in the questionnaire once. All data in this study were coded, no personal information of the participants was involved, and only the statistical analysts could access the original data. A total of 524 questionnaires were collected, and 8 questionnaires with all consistent answers were excluded. Finally, 516 valid questionnaires were analyzed, with an effective recovery rate of 98.5%.

#### 2.3.3 Statistical methods

SPSS 23.0 statistical software was used for data analysis. Measurement data were described using mean ± standard deviation, and count data were described using the number of cases and percentage. Pearson correlation analysis and multiple linear regression analysis were used to explore the relationships among the three variables. The PROCESS plug-in 4 was used for mediating effect analysis. The Bootstrap method was used for 5,000 repeated samplings to calculate the 95% confidence interval (CI). If the results did not include 0, the mediating effect was significant. A *p*-value <0.05 was considered statistically significant.

## 3 Results

### 3.1 General information of the participants

A total of 516 clinical nurses were included in this study, with an average age of 33.74 ± 6.95 years. Among them, 47 were male (9.1%) and 469 were female (90.9%); in terms of marital status, 110 were unmarried (21.3%) and 406 were married (78.7%); regarding professional titles, 248 had primary titles (48.1%), 234 had intermediate titles (45.3%), and 34 had senior titles (6.6%); in terms of employment type, 100 were regular nurses (19.4%) and 416 were non-regular nurses (80.6%); regarding educational background, 79 had a junior college degree (15.3%), 413 had a bachelor’s degree (80.0%), and 24 had a postgraduate degree or above (4.7%); in terms of average monthly income, 158 earned <5,000 yuan (30.6%), 169 earned 5,001–10,000 yuan (32.8%), and 189 earned >10,000 yuan (36.6%); regarding the number of night shifts per week, 216 had ≤1 shift (41.9%), 188 had 2 shifts (36.4%), and 112 had ≥3 shifts (21.7%); in terms of weekly working hours, 141 worked ≤8 h (27.3%), 253 worked 8–10 h (49.0%), and 122 worked >10 h (23.7%); regarding whether there were tasks beyond clinical work, 355 answered “yes” (68.8%) and 161 answered “no” (31.2%).

### 3.2 Scores of turnover intention, psychological contract breach, and illegitimate tasks among nurses

The results of this study showed that the score of turnover intention among clinical nurses was (13.14 ± 4.15), with an average item score of (2.19 ± 0.69). Among them, 34 nurses (6.6%) had very low turnover intention, 327 (63.4%) had low turnover intention, 84 (16.3%) had high turnover intention, and 71 (13.7%) had very high turnover intention. The score of psychological contract breach was (55.79 ± 15.50), and the score of illegitimate tasks was (29.06 ± 8.69) (see Table 1).

**Table 1:** Scores of turnover intention, psychological contract breach, and illegitimate tasks among nurses (*n* = 516).

Item	Number of items	Score range	Score (mean ± SD)	Score rate (%)
Turnover intention	6	6 ~ 24	13.14 ± 4.15	54.8%
Turnover intention I	2	2 ~ 8	4.41 ± 1.46	55.1%
Turnover intention II	2	2 ~ 8	4.34 ± 1.38	54.3%
Turnover intention III	2	2 ~ 8	4.40 ± 1.42	55.0%
Psychological contract breach	20	20 ~ 100	55.79 ± 15.50	55.8%
Developmental responsibility	6	6 ~ 30	14.54 ± 5.35	48.5%
Team responsibility	8	8 ~ 40	23.69 ± 6.47	59.2%
Realistic responsibility	6	6 ~ 30	17.56 ± 6.00	58.5%
Illegitimate tasks	8	8 ~ 40	29.06 ± 8.69	72.7%
Unreasonable tasks	4	4 ~ 20	14.66 ± 4.59	73.3%
Unnecessary tasks	4	4 ~ 20	14.40 ± 4.34	82.0%

### 3.3 Correlations among scores of turnover intention, psychological contract breach, and illegitimate tasks among nurses

The results of this study showed that the total score of illegitimate tasks and its dimensions were positively correlated with the total score of psychological contract breach and its dimensions (*r* = 0.401–0.623, *p* < 0.01), and also positively correlated with the total score of turnover intention and its dimensions (*r* = 0.446–0.495, *p* < 0.01). The total score of psychological contract breach and its dimensions were positively correlated with the total score of turnover intention and its dimensions (*r* = 0.439–0.733, *p* < 0.01) (see Table 2).

**Table 2:** Correlations among scores of turnover intention, psychological contract breach, and illegitimate tasks among nurses (*n*, *r*).

Item	1	2	3	4	5	6	7	8	9	10	11
Turnover intention (1)	1										
Turnover intention I (2)	0.977^**^	1									
Turnover intention II (3)	0.971^**^	0.923^**^	1								
Turnover intention III (4)	0.976^**^	0.934^**^	0.921^**^	1							
Psychological contract breach (5)	0.623^**^	0.610^**^	0.614^**^	0.600^**^	1						
Developmental responsibility (6)	0.733^**^	0.716^**^	0.722^**^	0.704^**^	0.782^**^	1					
Team responsibility (7)	0.458^**^	0.448^**^	0.453^**^	0.439^**^	0.915^**^	0.556^**^	1				
Realistic responsibility (8)	0.464^**^	0.453^**^	0.455^**^	0.449^**^	0.900^**^	0.531^**^	0.790^**^	1			
Illegitimate tasks (9)	0.495^**^	0.469^**^	0.493^**^	0.486^**^	0.623^**^	0.432^**^	0.612^**^	0.563^**^	1		
Unreasonable tasks (10)	0.487^**^	0.466^**^	0.481^**^	0.478^**^	0.618^**^	0.439^**^	0.604^**^	0.554^**^	0.975^**^	1	
Unnecessary tasks (11)	0.476^**^	0.446^**^	0.479^**^	0.468^**^	0.593^**^	0.401^**^	0.588^**^	0.542^**^	0.972^**^	0.895^**^	1

**p < 0.01.

### 3.4 Mediating role of psychological contract breach between illegitimate tasks and turnover intention among nurses

Taking the score of illegitimate tasks as the independent variable (*X*), the score of psychological contract breach as the mediating variable (*M*), and the score of turnover intention as the dependent variable (*Y*), the PROCESS plug-in model 4 developed by Hayes was used to test the mediating effect. The results showed that the score of illegitimate tasks could positively predict the score of psychological contract breach and the score of turnover intention, and the score of psychological contract breach could positively predict the score of turnover intention (see Table 3).

**Table 3:** Regression models of the mediating role of psychological contract breach between illegitimate tasks and turnover intention among nurses.

Regression equation		Fit indices			Significance of regression coefficients	
Outcome variable	Predictor variable	*R*	*R* ^2^	*F*	*β*	*t*
Psychological contract breach	Illegitimate tasks	0.623	0.388	325.203	0.825	12.9018.033^**^
Turnover intention	Illegitimate tasks	0.638	0.407	176.142	0.083	4.009^**^
	Psychological contract breach				0.185	11.854^**^

**p < 0.01.

**Figure 1:**
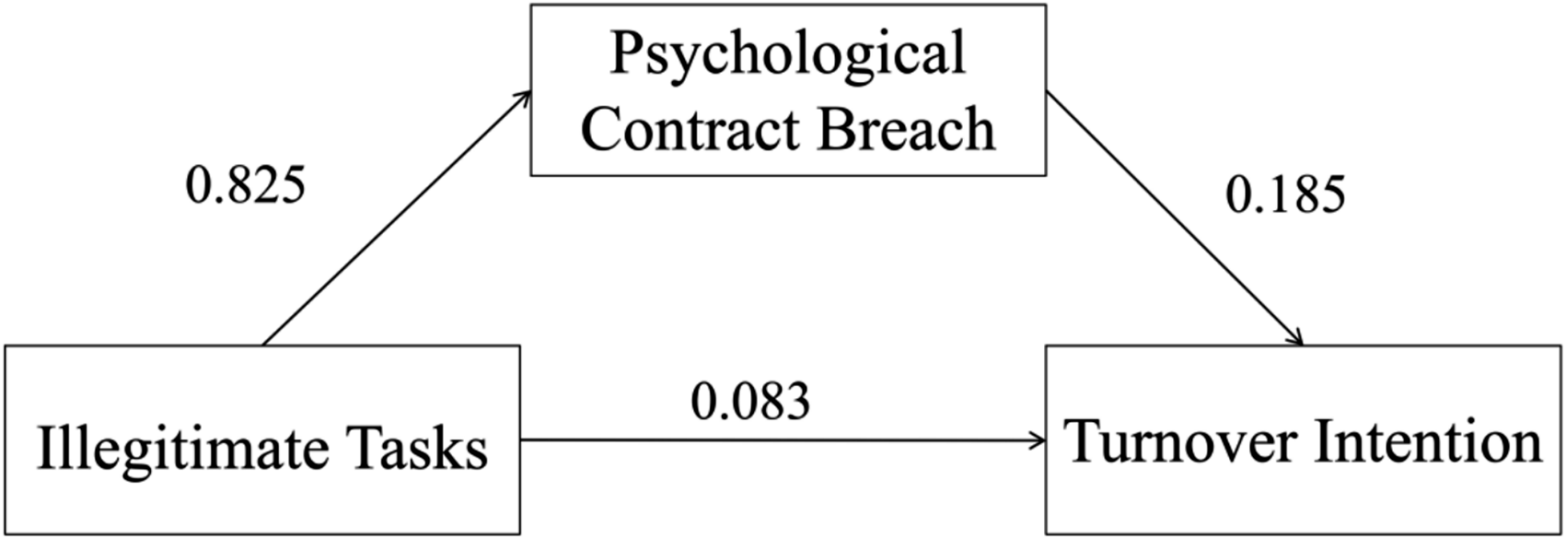
The mediating model of psychological contract breach between illegitimate tasks and turnover intention among nurses.

The Bootstrap method was used for 5,000 repeated samplings to test the constructed mediating effect, and the mediating model is shown in Figure 1. The results showed that the 95% confidence interval of the mediating effect did not include 0, indicating that psychological contract breach played a significant mediating role between illegitimate tasks and turnover intention. The mediating effect value was 0.825 × 0.185 = 0.153. Meanwhile, the 95% confidence interval of the direct effect of psychological contract breach on turnover intention also did not include 0, indicating that the direct effect was significant, with an effect value of 0.083. The total effect = mediating effect + direct effect = 0.236, and the mediating effect accounted for 64.8% of the total effect (see Table 4).

**Table 4:** Analysis of the mediating role of psychological contract breach between illegitimate tasks and turnover intention among nurses.

Path	Standardized path effect	Effect size (%)	95% CI	
			Lower limit	Upper limit
Direct effect: Illegitimate tasks → Turnover intention	0.083	35.2	0.042	0.124
Mediating effect: Illegitimate tasks → Psychological contract breach → Turnover intention	0.153	64.8	0.114	0.195
Total effect: Illegitimate tasks → Turnover intention	0.236	100	0.200	0.272

## 4 Discussion

This study explored the relationship between illegitimate tasks and turnover intention among nurses, with a focus on the mediating role of psychological contract breach. The study found that illegitimate tasks have a negative impact on turnover intention, psychological contract breach is an important risk factor for turnover intention, and psychological contract breach plays a partial mediating role between illegitimate tasks and turnover intention.

Turnover intention is an important antecedent variable of turnover behavior and the most effective indicator for predicting turnover behavior (Dall’Ora et al., 2020). The results of this study showed that the score of turnover intention among clinical nurses was (13.14 ± 4.15), and 30% of the nurses had high or very high turnover intention, which was basically consistent with the research results of Cao et al. (2021). A survey in the United States showed that before 2020, 48% of nurses considered leaving clinical practice and finding jobs outside the medical industry (Medscape, 2020). The high turnover intention and large-scale loss of nurses have affected the development of the medical industry and increased potential risks to nursing safety in medical services. The reasons for this may be as follows: first, under the influence of the traditional concept of “valuing medicine over nursing,” current nursing staff face problems such as low professional value and recognition, relatively low income, frequent night shifts, and heavy workload, resulting in an overall low level of work well-being. Second, Chinese nurses, as a team dominated by women, not only have to undertake heavy nursing tasks but also take on the responsibility of caring for children and families, leading to prominent conflicts between work and family balance and affecting the stability of their careers (Han et al., 2021). Male nurses, due to gender discrimination, are more prone to having the tendency to resign. In addition, with the development of the economy and society, the demand for health-related industries has been increasing. As important health promoters, nurses have more employment options and more opportunities to engage in other health industries, which to a certain extent increases their turnover intention. Therefore, it is suggested that nursing managers should pay attention to nurses’ physical and mental health, balance the relationship between work and family, rationally allocate nursing human resources, optimize nurses’ work performance, and thus improve the current situation of high nurse turnover rate.

A psychological contract refers to the subjective belief of the mutual exchange conditions between employees and the organization in the employment relationship. When individuals believe that the organization has not fulfilled its due reciprocal commitments, psychological contract breach occurs, which shakes their organizational beliefs and generates negative emotions and behaviors. The results of this survey showed that the level of psychological contract breach among nurses was moderate, which was consistent with the study of Trybou et al. (2016). The reasons for this phenomenon may be as follows: first, at the individual level, with the increase of working years, nurses’ expectations for promotion, training, and professional growth continue to increase. If their academic qualifications and abilities are not fully valued or utilized, they are prone to a sense of “being wasted,” leading to psychological contract breach (Elangovan et al., 2021). Second, organizational support is an important protective factor for nurses’ professional identity, while the lack of organizational justice is considered to have the greatest impact on nurses’ psychological contract breach (Zinta et al., 2008). If nurses feel that the organization does not support their work and lacks authorization to participate in decision-making, they are prone to a sense of “being ignored,” which further leads to psychological contract breach. In addition, due to the serious shortage of nurses, nurses generally face problems such as insufficient staffing, fast work rhythm, heavy tasks, high frequency of night shifts, and no compensation for overtime work, which makes it impossible for them to provide care in accordance with professional standards and generates a sense of frustration of “being unable to be competent in the professional role,” thus triggering psychological contract breach (Elangovan et al., 2021). Therefore, it is suggested that nursing managers should start from various aspects such as improving the sense of organizational support, optimizing the management system, improving the professional environment, and enhancing communication and respect, to build a healthier and more sustainable nursing human resource ecosystem and narrow the gap between nurses’ expectations and reality.

The results of this study showed that the level of illegitimate tasks among clinical nurses was relatively high, especially unnecessary tasks, which was consistent with the research results of Wei et al. (2025). Studies have shown that nurses’ frequent performance of illegitimate tasks conflicts with their work cognitive schema, which increases their psychological pressure, negative emotional experience, and work resource depletion (Guan et al., 2025). This may be related to the following reasons: on the one hand, nurses generally face the problem of shortage of staff and heavy workload, which forces them to take on non-nursing responsibilities. A large number of non-nursing responsibilities disrupt the performance of their established responsibilities and affect the performance of their original work (Kilponen et al., 2021). On the other hand, the nursing profession has a relatively low status in society and the medical system, and the sense of professional identity is not strong, which makes employees bear more “low-value” or “marginalized” tasks, further inducing other more negative results (Kilponen et al., 2021). In addition, the medical system values medical treatment over nursing, and resource allocation is biased toward doctors. Nursing work is systematically underestimated, making nurses a “fill-in” role, which is considered a special form of unfair treatment in the organization. This makes employees experience high levels of role ambiguity, role conflict, and other pressures, and generates negative results because they cannot feel the importance and necessity of their work (Kilponen et al., 2021). Therefore, nursing managers need to systematically intervene from aspects such as clarifying job responsibilities, optimizing resource allocation, improving the status of nursing, and improving the organizational culture to reduce the level of illegitimate tasks.

This study revealed through correlation analysis that there is a significant positive correlation among illegitimate tasks, psychological contract breach, and turnover intention of nurses. The results showed that illegitimate tasks of nurses are positively correlated with turnover intention (Hypothesis 1 verified), which supports the hypothesis of a vicious circle between illegitimate tasks and high nurse turnover rate, and is consistent with the research results of Li et al. (2023). Specifically, illegitimate tasks often mean high input and low recognition. When nurses are required to complete tasks outside their job scope, meaningless or low-value tasks, they are prone to the cognition that “efforts do not get the due reward,” and this sense of imbalance will significantly increase their turnover intention. In addition, as an “identity-offensive stressor,” illegitimate tasks will weaken nurses’ sense of professional identity and induce burnout symptoms such as emotional exhaustion, cynicism, and reduced sense of accomplishment, thereby weakening their identification with the organization. The Social Exchange Theory also points out that in the process of interaction with the organization, employees will strive to maintain the balance of the exchange relationship. When this balance is broken, nurses will have role expectation conflicts, which easily lead to expectation gaps and thus generate the idea of leaving (Homans, 1958).

The results of this study showed that psychological contract breach of nurses is positively correlated with turnover intention (Hypothesis 2 verified), which supports that psychological contract breach is an important predictor of turnover intention, and is consistent with the research results of Qian et al. (2015). The Social Cognitive Theory holds that individuals’ cognition affects their behavior decisions. When they perceive psychological contract breach, employees cannot have sufficient trust in the organization, that is, they cannot determine whether participating in knowledge exchange can obtain equivalent returns, so they may refuse to respond to the organization’s knowledge requests. Second, because their own interests are not met, psychological contract breach is likely to trigger negative emotions of employees, affect their organizational commitment and job satisfaction in daily work, and thus reduce the belief of reciprocal commitment between nurses and the organization. In addition, as a chronic stressor, psychological contract breach will aggravate burnout symptoms such as emotional exhaustion and depersonalization, weaken their professional identity and work engagement (Sheehan et al., 2019), and thus increase turnover intention.

This study also found that illegitimate tasks of nurses are positively correlated with psychological contract breach (Hypothesis 3 verified), which supports that the increase of illegitimate tasks will consume the contractual relationship between nurses and the organization, and is consistent with the research results of Ding and Kuvaas (2023). According to the Stress-as-Offense-to-Self Theory (Semmer et al., 2019), when employees are forced to perform illegitimate tasks, their psychological contracts will conflict due to the lack of organizational responsibility, leading to psychological contract breach. First, illegitimate tasks are often regarded as a manifestation of unfair distribution, especially when other positions do not need to undertake similar tasks, this sense of unfairness will directly damage nurses’ psychological contract of “fair treatment” by the organization, leading to contract breach. Second, as a chronic stressor (Metin et al., 2016), illegitimate tasks will trigger negative emotions such as anger, burnout, and powerlessness among nurses, weaken their emotional trust in the organization, make them more likely to interpret the organization’s behaviors as “breaking promises,” and thus reduce nurses’ organizational trust. This study reveals the correlation and influence among illegitimate tasks, psychological contract breach, and turnover intention, suggesting that nursing managers should reduce illegitimate tasks and strengthen organizational respect and support, which are key strategies to maintain nurses’ psychological contracts and stabilize the nursing team.

The mediating effect analysis of this study showed that psychological contract breach plays a mediating role between illegitimate tasks and turnover intention of clinical nurses, and the mediating effect accounts for 64.8% of the total effect (Hypothesis 4 verified). That is, illegitimate tasks directly affect turnover intention and indirectly affect turnover intention through psychological contract breach. On the one hand, illegitimate tasks mean that nurses not only need to complete their own work but also spend time and energy on work that is not originally theirs, increasing the cost of work input, making it impossible for them to obtain expected returns and develop in coordination with the organization (Wang et al., 2018). On the other hand, when nurses take on illegitimate tasks, these tasks beyond the scope of their duties will make nurses think that the organization has not fulfilled the contract, or feel that their input and return are not equal, thus triggering the negative emotional experience of psychological contract breach. Once this emotion is triggered, it will show resistance to the current work (Morrison and Robinson, 1997). Second, when employees experience psychological contract violation in the workplace for a long time, their sense of responsibility to the organization will also decrease, and they will be more likely to think that the development of the organization has nothing to do with themselves, thus generating turnover intention. The study by Turnley and Feldman (1999) also pointed out that when employees experience psychological contract violation, it will have a series of impacts on their work attitudes, thereby affecting their career stability. Some studies also pointed out that as a new stressor, illegitimate tasks will not only threaten employees’ professional identity and role cognition but also make them feel more work pressure, trigger emotions of anger and resentment among employees, reduce job satisfaction, and generate turnover intention (Van Schie et al., 2014). Therefore, nursing managers should pay attention to the negative impact of illegitimate tasks on the turnover intention of clinical nurses. They should actively create a good organizational atmosphere, strengthen the connotation and value significance of nursing professionalism, and enhance nurses’ hope and motivation for career development. In addition, focus on the mental health of new nurses, improve their psychological quality and stress response ability, guide and help nurses correctly understand and actively respond to difficulties in the process of career development, thereby maintaining psychological contracts and reducing the nurse turnover rate.

## 5 Limitations

This study confirms the relationship among illegitimate tasks, psychological contract breach, and turnover intention of clinical nurses, and provides a theoretical basis for reducing the nurse turnover rate. However, there are also some limitations: (1) Regional limitation: Samples were only from Sichuan Province, limiting generalizability to other regions (e.g., eastern vs. western China with different economic levels). Future studies should include nurses from different provinces to consider economic and cultural differences. (2) Cross-sectional design: Cannot establish causal relationships—for example, we cannot confirm that illegitimate tasks “cause” psychological contract breach (reverse causality is possible: nurses with high contract breach may perceive more tasks as illegitimate). Longitudinal studies are needed to track changes in variables over time (e.g., 6 month follow-ups). (3) Sampling method: Convenience sampling may introduce selection bias (e.g., over-representing nurses from cooperative hospitals). Stratified random sampling across hospital levels and regions is recommended for future research. (4) Potential confounding variables: Did not control for variables such as job burnout or social support, which may affect the model. Future studies should include these variables to improve model accuracy.

## 6 Conclusion

The results of this study show that the turnover intention of clinical nurses is at a relatively high level. Illegitimate tasks have a significant positive predictive effect on nurses’ turnover intention among Chinese clinical nurses, and psychological contract breach plays a partial mediating role between nurses’ illegitimate tasks and turnover intention. This model clarifies how nurses’ illegitimate tasks affect turnover intention (the mediating role of psychological contract breach) and provides a theoretical basis for nursing managers to reduce the nurse turnover rate. The implications of this study are mainly as follows: (1) Nursing managers should pay attention to illegitimate tasks and their negative consequences, continuously improve the standardization of work design and the clarity of responsibilities, reasonably divide tasks, and minimize the assignment of illegitimate tasks. (2) Nursing managers need to actively establish and reshape the psychological contracts of employees. In the context of local culture, employees are more dependent on verbal commitments between the two parties and mutually agreed relationships. Therefore, medical institutions should establish a true and reasonable contractual relationship with employees to increase employees’ positive beliefs and commitments to the organization. (3) Nursing managers need to establish a management method conducive to the healthy development of the nursing team, pay sufficient attention to employees, focus on communication with employees when assigning tasks, take employees’ work feedback seriously, and establish a good working atmosphere.

## Data Availability

The raw data supporting the conclusions of this article will be made available by the authors, without undue reservation.
